# The role of pulmonary rehabilitation in severe asthma: a comprehensive review

**DOI:** 10.3389/fmed.2025.1709710

**Published:** 2025-10-28

**Authors:** Gabriella Guarnieri, Michela Pozza, Andrea Vianello

**Affiliations:** Respiratory Pathophysiology Unit, Department of Cardiac-Thoracic-Vascular Sciences and Public Health, University of Padova, Padova, Italy

**Keywords:** aerobic training, inspiratory muscle training, breathing retraining, telerehabilitation, maintenance strategies

## Abstract

**Background:**

Severe asthma remains a major problem despite pharmacological advances. Pulmonary rehabilitation (PR) is established in chronic respiratory disease but its role in severe asthma is unclear.

**Objectives:**

Summarise evidence on PR in severe and uncontrolled asthma, describe PR-modalities, and outline implementation and research priorities.

**Methods:**

Narrative review of systematic reviews and clinical studies of multidimensional PR programmes and isolated components [aerobic training, inspiratory muscle training (IMT), breathing retraining, neuromuscular electrical stimulation (NMES), telerehabilitation]. Outcomes included asthma control, HRQoL, exercise capacity and healthcare utilisation.

**Results:**

Multicomponent PR improves exercise capacity and multiple QoL domains; pooled data show substantial increases in six-minute walk distance. Combined exercise, education and self-management produced clinically meaningful improvements in asthma control and symptoms, notably patients with uncontrolled disease and functional impairment. IMT, NMES and breathing retraining improved inspiratory strength, peripheral muscle function and hyperventilation symptoms. Telerehabilitation expands access but requires attention to digital literacy and adherence. Heterogeneity, small samples and attrition limit generalisability.

**Conclusion:**

PR is a promising personalised, multidisciplinary adjunct for severe asthma. Larger phenotype-stratified trials, harmonised outcome sets and implementation research are needed to define candidate selection, optimal dose and cost-effectiveness; embedding PR within severe asthma centres may optimise outcomes and reduce healthcare use.

## 1 Introduction

Asthma is a chronic inflammatory disease of the airways, characterized by variable airflow obstruction, bronchial hyperresponsiveness and recurrent respiratory symptoms (1). It affects an estimated 339 million people worldwide and imposes a substantial public-health and socio-economic burden (2). Despite therapeutic advances, including inhaled corticosteroids, long-acting bronchodilators and targeted biologic agents, a proportion of patients continue to experience severe or uncontrolled asthma according to ATS/ERS definitions (3). Difficult-to-treat asthma refers to asthma that remains uncontrolled despite medium- or high-dose inhaled corticosteroids with a second controller, or requires oral corticosteroids to achieve control (1, 3). It is distinguished from severe asthma, a subset in which disease remains uncontrolled despite optimized therapy and management of comorbidities, or worsens when treatment is stepped down. In clinical practice, poor adherence to therapy, incorrect inhaler technique and underuse of maintenance inhaled corticosteroids contribute to disease instability and recurrent exacerbation cycles. Multimorbidity is common in severe asthma; rhinosinusitis, obesity, gastroesophageal reflux, anxiety, depression and dysfunctional behaviours amplify clinical complexity, worsen control and increase healthcare utilisation (4, 5). A comprehensive, multimodal care strategy is therefore required to optimise clinical trajectories for these patients (1). Current guidelines recommend personalised care plans negotiated between patients, caregivers and the multidisciplinary clinical team, within a continuous cycle of assessment, adjustment and review (1). Biologic therapies targeting specific inflammatory pathways have improved outcomes in selected phenotypes, but residual symptom burden and functional impairment persist in many patients, motivating investigation of non-pharmacological adjuncts. In other chronic respiratory diseases, notably COPD, pulmonary rehabilitation (PR) is an evidence-based cornerstone that improves dyspnoea, fatigue, emotional function, quality of life and exercise capacity; telemedicine delivery is an emerging method to increase access (6, 7). This review evaluates PR in severe and uncontrolled asthma, analyses available evidence, describes modalities and practical aspects and identifies research priorities.

## 2 Pulmonary rehabilitation

Pulmonary rehabilitation (PR) is defined by ATS/ERS as a comprehensive intervention based on detailed patient assessment, followed by individually tailored therapies including exercise training, education and behaviour change to improve physical and psychological condition and to promote long-term adherence to health-enhancing behaviours (8). Despite early consensus statements and efficacy data, access to PR remains limited by insufficient funding, restricted dedicated resources, variable awareness among clinicians and patients, and inconsistent training opportunities for providers; its availability differs widely across countries (9).

GINA recommendations recognises PR as complementary to pharmacological management (level A evidence for improvements in functional exercise capacity and health-related quality of life), although direct evidence for symptom control and long-term durability in asthma is less conclusive (1). PR programmes are multimodal by design and should be adapted to individual needs and comorbidities (9).

### 2.1 Components and duration of PR programme

Most high-quality data relative to PR programme derive from COPD populations, but practical principles could be at least partially translated to asthma. Meta-analyses and consensus statements recommend a minimum programme duration of approximately eight weeks to achieve clinically meaningful gains, with outpatient models typically delivering two to three supervised sessions per week and inpatient models offering more frequent supervised training (6, 9). A combination of supervised sessions and prescribed home exercise, supported by follow-up and booster cycles, promotes maintenance of benefits; repeated programme cycles produce effects comparable to initial one (9). For asthma patients, no consensus yet exists regarding optimal duration, total session number, intensity schema, or staffing composition—which may include physicians, healthcare professionals, psychologists, nutritionists, and social workers—as heterogeneity in study designs limits generalisability (8). High-quality programmes should include structured supervised exercise, education and behavioural support, comprehensive assessment and personalised outcome measurement, and home-exercise prescription with encouragement to perform daily physical activity (9). Exercise sessions typically last 30–90 min, two to three times per week, and may combine aerobic training (e.g., cycling or treadmill) with strength and flexibility exercises, either with equipment or bodyweight (10, 11). Educational components typically cover safety, treatment use, exacerbation management, and lifestyle aspects such as physical activity, nutrition, emotional well-being, and social interaction (12).

## 3 Current evidence on pulmonary rehabilitation in severe asthma

Two recent systematic reviews provide complementary perspectives. A Cochrane review of comprehensive PR programmes reported significant improvement in functional capacity, with a pooled mean increase of 79.8 meters in the six-minute walk test (6MWT), while improvements in maximal exercise capacity (VO₂max, VO₂peak) were modest and uncertain due to heterogeneity and limited long-term data (13). Zampogna et al. (14) considered isolated rehabilitation components (aerobic training, inspiratory muscle training, breathing exercises, yoga, education) and found uncertain or limited effects on health-related quality of life (Asthma Quality of Life Questionnaire AQLQ) and asthma control, reflecting methodological diversity across studies.

Studies targeting severe or uncontrolled asthma show promising but heterogeneous results. Majd et al. reported that a 12-week tailored rehabilitation programme yielded improvements in physical performance, health-related quality of life and asthma control in patients with severe disease (15). Schultz et al. evaluated a 3-week inpatient PR programme in patients with Asthma Control Test (ACT) scores <20 and documented clinically meaningful ACT improvements and secondary benefits in AQLQ, SGRQ (St. George’s Respiratory Questionnaire), core respiratory symptoms, self-management skills, illness perception, anxiety and depression; many improvements persisted up to 12 months (16). Ricketts et al. (17) studied an 8-week programme for patients with uncontrolled asthma and BMI ≥ 25 kg·m^−2^ (Body mass index) combining one supervised weekly session with two home sessions. The intervention improved asthma control, symptoms and exercise tolerance, particularly in activity and symptom domains of the AQLQ, but did not increase accelerometer-measured physical activity, suggesting limited behavioural change. A 12-month follow-up of the cohort demonstrated sustained improvements among completers but highlighted high attrition and barriers including the COVID-19 pandemic, work and personal constraints, and perceived exercise difficulty (18). A retrospective home-based PR analysis showed improved 6MST (six-minute stepper test) step counts and long-term quality of life in severe asthma patients, though anxiety and depression changes were less evident compared with COPD cohorts (19).

In summary these data indicate that PR confers functional, symptomatic and quality-of-life benefits in selected asthma populations, especially those with uncontrolled symptoms or deconditioning; however, small sample sizes, heterogeneous interventions and retention challenges limit definitive conclusions.

## 4 Strategic approaches and modalities in PR in severe asthma

The most relevant methods utilized for pulmonary rehabilitation in asthma have been summarized and listed in Table 1 and illustrated in the paragraphs below.

**Table 1:** Evidence of major PR methods in asthma, with particular attention to the type of interventions, programs and outcomes.

Study	Type of study	Population	Intervention	Programs	Outcomes
McLoughlin et al. (11)	Systematic review and meta-analysis	Uncontrolled, moderate to severe asthma Population (n): 176 Adults Number of studies: 4	Exercise training	-Period: ⩾2 weeks -Frequency: range 2–5 times per week, with a duration of 30 to 60 min -Physical activities: aerobic and low-intensity exercise (i.e., yoga), walking, running, cycling, swimming, weight-bearing exercise, pulmonary rehabilitation and interval training. Movement-based interventions, interventions with wearable technology. -Vary greatly in frequency, intensity, duration, and type of activity -Additional educational and/or nutritional interventions, psychological sessions.	Physical activity: overall significant improvement in steps per day in favor of the physical activity intervention. Effects varied by study, with largest benefits seen in pedometer-based programmes; improvements often not sustained post-intervention. Health-related quality of life: AQLQ: overall positive effect of physical activity (not clinically significant). Asthma control: Asthma control (ACQ, symptom-free days, exacerbations): significant ACQ improvement; no overall effect on symptom-free days; exacerbation benefit in one study only. Lung function: only 2 studies examined lung function (improvement in FRC and ERV). Improvement in FVC and FEV1 (1 study). Systemic and airway inflammation: no significant within or between group differences in airway inflammation. Health-related outcomes: in favor of intervention group (where reported) for BMI, fat mass, and waist circumference and muscle strength. Significant improvement in exercise capacity large positive effect on VO₂max. No statistically significant benefits in anxiety or depression measured with HADS.
Kuder et al. (21)	Systematic review	Asthma Adults Number of studies: 35 publications from 20 studies	Physical activity	Interventions: varied across studies (walking or running; circuit training or other aerobic and resistance exercises). In 9 studies there are supplementary intervention ranged from breathing exercises, a weight loss or dietary component or vitamin supplementation. Supervision: 9 studies Frequency: from daily to once weekly Duration: varied from six weeks to one year.	Lung Function (spirometry measurement): improvement in at least one measure (6 studies), i.e., FVC, FEV1, FEV1/FVC. No between-group differences in any measure (1 studies). Asthma control: measured with ACQ, rescue inhaler uses, or emergency room visits or daily diary symptom reports or number of reported asthma exacerbations (12 studies): 7 studies showed a statistically significant improvement in at least one outcome measure of asthma control. Health Care Related Quality of Life: 11 studies. AQLQ or mini-AQLQ (9 studies): 5 showed a statistically significant improvement at least in one domain. Half of the studies found some improvement in quality of life. Serologic Inflammatory Markers: Significant reduction in at least 1 inflammatory (i.e., IL-6) (3 studies). No significant between-group differences in any inflammatory marker for three studies.
Hansen et al. (22)	Systematic review and meta-analysis	Asthma mild to moderate persistent (2), moderate to severe persistent (6) Population (n): 543 (475 at the follow-up) Adults Number of studies: 11	Aerobic exercise	Duration: 8–12 weeks Intervention: supervised (9) and unsupervised (2 studies). Cycling, treadmill, walking, mixed aerobic exercise. Exercise intensity: 60–75% of HRmax (7 studies). HIIT at >90% HRmax.	Asthma control: ACQ (5 studies), HRQOL (2 studies): difference in favor of exercise training, but with high heterogeneity Lung function: of the ten studies reporting lung function via FEV1, a difference in favor of exercise training was observed, but with considerable heterogeneity across studies. Airway inflammation: Overall, no difference was found in the SMD for airway inflammation with considerable heterogeneity across studies.
Carson et al. (10)	Systematic review and meta-analysis	Asthma Population (age): >8 years Number of studies: 21 (13 for quantitative analysis)	Exercise training	Activity: Physical training (whole body aerobic exercise) lasting for at least 20 to 30 min, two to three times a week, with a minimum duration of four weeks. The length of the physical training programs varied from 6 to 16 weeks	Asthma symptoms: No significant difference in asthma control was found between intervention and control groups reported improvements in symptom-free days for the intervention groups compared to controls (3 studies) FEV1: Pooled data (9 studies) reported no significant effect of physical training on FEV1. FVC: pooled results showed that physical training had no effect on FVC (7 studies). VEmax, VO_2_ max: VEmax in 5 studies reported no significant improvement. VO_2_max: 8 studies, significant improvement (clinical and statistical). Significant work capacity (2 studies). HRmax: Four studies (81 participants) were pooled, showing a significant increase in HRmax (high heterogeneity) MVV: improvement in pre-post exercise (1 study). 6MWD: 1 study of 34 people. Increased the distance, but no statistical significance between groups. QoL: An improvement in quality of life (QoL) is observed in the intervention groups. However, clinical and statistical significance is not present in all studies.
Valkenborghs et al. (24)	Randomized controlled trial	Asthma and current episodic symptoms, BMI ≤ kg/m^2^ Population (n): 46 Adults (18–55)	Exercise training	Activity: (1) moderate-intensity exercise 3/week for 12 weeks for 45 min each (2) Vigorous-intensity exercise 3 times per week for 12 weeks for 30 min each (3) Control group, participants maintain their current physical activity levels for 12 weeks Duration: 12 weeks	Quality of life (AQLQ): (1) Clinically significant change (mean:0.63, *p* < 0.001) in IG vs. CG in all subdomains (2) Statistically (non clinical) significant change in symptoms and enviromental stimuli. Asthma Control (ACQ): (1) Statistically and clinical significant change (mean −0.51, *p* = 0.003) (2) Statistically (non clinical) significant change Lung function: no changes both in moderate or vigorous. Cardiorespiratory fitness and body composition: (1) non-significant improvement in relative VO₂peak; significant reduction in gynoid fat mass (2) statistically significant improvement in relative and absolute VO₂peak; trend toward reducing both android and gynoid fat mass Airway and systemic inflammation: (1) Resulted in a significant reduction in sputum macrophage (−1341*10^4^/mL, *p* = 0.024) and lymphocyte counts (−114*104/mL, *p* = 0.036) compared with the control group, and a reduction in macrophages (−1791*10^4^/mL, *p* = 0.002) compared with the vigorous-intensity group. (2) Did not produce significant changes in airway or systemic inflammatory markers. Associations between changes in fitness and body composition with changes in asthma outcomes: In the cohort, reductions in total, android, and gynoid fat mass were associated with significant improvements in asthma-related quality of life, particularly in the symptoms domain, and with favorable inflammatory changes, whereas increased VO₂peak was only linked to higher plasma IL-1ra levels. No other associations between fitness gains and asthma outcomes were observed.
Lista-Paz et al. (31)	Systematic review and meta-analysis	Mild to moderate asthma, moderate-to-severe asthma (1 study) Population (n): 27 Adults and children Number of studies: 11 (1 with children population)	Inspiratory muscle training (IMT)	Period: 3 weeks to 6 months Frequency: from 2 to 7 days, from 10 to 30 min/day Activity: training load anging from 15 to 80% of PImax; in some studies, it was between 40 and 50%, with progression based on PImax reassessment Repetitions and frequency: varied between studies	Respiratory muscle strength and endurance: consistently improved PImax across studies (MD: 21.95 cmH2O [95%CI 15.05; 28.85] and overall effect Z = 6.23 [*p* < 0.01)(I2 = 85%)], had no significant effect on PEmax, and improved respiratory muscle endurance in the few studies that measured it. Use of rescue medication (4 studies): significant decrease in puff/day of b2-agonist in the IMT group (3 studies) Asthma-related symptoms and asthma control (9 studies): IMT appears to improve symptoms, dyspnoea, and fatigue in several studies; however, data are not uniform, and none have directly assessed overall asthma control using validated instruments. Lung function: no significant differences in PEF, FEV1 and FVC (5 studies) Exercise capacity: (6MWT: 2 studies) (ISWT:1 study): no significant differences between groups, but with high heterogeneity. Statistical differences in studies with load > 50% PImax and duration > 6 weeks Number of emergency department visits and hospital admissions (3 studies): significant decrease in 1 study Health-related quality of life: significantly differences in pre-post intervention at AQLQ or St. George’s Respiratory Questionnaire (2 studies)
Sogard et al. (30)	Narrative Review	Asthma with different severity Number of studies: 20 Adult and children	Inspiratory muscle training (IMT)	Duration: varied across studies form 3 weeks to 6 months Activity: flow-resistive, standard protocols, use of device with different resistance Frequency and duration: varied across studies.	Pulmonary function: studies showed mixed effects on dynamic lung volumes, with improvements more often seen when pressure-threshold devices are used, likely influenced by training protocols, duration, asthma severity, and muscle adaptations increasing thoracic expansion. Respiratory muscle function: IMT increases inspiratory muscle strength (and sometimes endurance) in asthma through structural and metabolic muscle adaptations, with combined IMT and exercise training giving the largest benefits. Medication use and Dyspnea: in high SABA users with asthma, IMT can strengthen inspiratory muscles, reduce breathlessness, and significantly cut reliever inhaler use, moving patients closer to safe usage levels and potentially lowering exacerbation risk. Asthma control, symptoms and Quality of life: IMT generally improves asthma control and quality of life in adults and children, though results vary across studies, and no trials assessed asthma severity via standardized airway challenge tests. Exercise tolerance and exertional dyspnea: IMT can improve walking and cycling performance, reduce dyspnoea and fatigue, lower exercise-related IL-6, and potentially enhance exercise tolerance in asthma by strengthening inspiratory muscles, reducing metaboreflex activation, and improving ventilatory efficiency.
Santino et al. (34)	Review	Mild to moderate asthma Population (n): 2880 Adults Number of studies: 22 (14 for quantitative analysis)	Breathing exercises	Activity: yoga or breathing exercise with different techniques.	Breathing exercise vs inactive control Quality of life: Breathing-based interventions generally improved asthma-related quality of life, with some studies reaching clinically meaningful changes, though effects varied and heterogeneity was high. Asthma symptoms and hyperventilation symptoms: Breathing-based interventions showed no clear benefit on ACQ-measured asthma control, but reduced hyperventilation symptoms in the medium term (4–6 months), with mixed results from other symptom measures. Number of acute exacerbations: Most studies reported fewer exacerbations after breathing-based interventions, but results were inconsistent. Physiological measures: Breathing-based interventions showed some short-term gains in % predicted FEV₁, PEFR, breathing rate, and end-tidal CO₂, but effects on absolute FEV₁, airway inflammation, and long-term outcomes were generally small or unclear. GP appointments: no clear difference in consultation rates (1 study) Breathing exercise vs asthma education Quality of life: B.E. improved asthma-related quality of life more than education, but benefits in were mostly seen after 4–6 months and were strongest in symptoms, activities, and emotional well-being (2 studies). Asthma symptoms: Breathing exercise interventions did not significantly improve asthma symptoms on ACQ (1 study), but they did reduce hyperventilation symptoms in the longer term (2 studies). Physiological measures: the intervention did not lead to clear improvements in either FEV₁ or end-tidal CO₂ levels compared with the control (1 study).
Xing et al. (20)	Network review	Asthma Population: 2062 Number of studies: 28	Breathing exercises + rehabilitative treatments + pharmacological care	Duration: from 1 week to 2 years, from 5 to 45 min. Activity: aerobic training, yoga, relaxation exercises, diaphragmatic exercises, breathing exercises.	FEV1: all exercise modalities improve FEV₁ in patients, but resistance training appears to have the greatest effect, while breathing exercises alone have the smallest. FVC: All exercise types improved FVC in patients, with combined breathing and aerobic training ranked most effective, and yoga the least. PEF: All exercise modalities improved PEF, with yoga ranked most effective and aerobic training the least. FEV1/FVC: Breathing training, aerobic training, and yoga all improved FEV₁/FVC, with breathing training ranked as the most effective.
Andreasson et al. (43) and protocol by Andreasson et al. (44)	Multicenter Randomized controlled trial	Uncontrolled asthma Population (n): 193 Adults	Breathing Exercise	Activity: Breathing exercises (BrEX) + Usual care (control group: only UC). The program involved a combination of the Buteyko and Papworth methods, with exercises aimed at modifying and normalizing the frequency and depth of breathing, using breathing modifications during exercise, and relaxing the shoulder girdle muscles and the thoracic and cervical areas. Informational material was provided, and the participants were trained in an exercise program to be performed for 10 min twice a day. Duration: three individual sessions lasting 60, 30, and 30 min, spaced 3–4 weeks apart. Data collection: 0–3–6-12 months	Quality of life (mini AQLQ): UC + BrEX produced a statistically significant improvement in MiniAQLQ scores compared with UC alone at 6 months, with consistent benefits at 3 and 12 months, although the odds of achieving a clinically meaningful change were not significant. Anxiety and depression (HADS): significant between-group difference at 6 months with a reduction in HADS-depression scores favoring UC + BrEX [−0.9 (21.67 to 20.14)] No differences in physiological outcomes or adverse event rates, and high adherence to the intervention (81% attended all sessions, 76% reported good/excellent home practice).
Zhang et al. (37)	Prospective single-center study	Severe, uncontrolled asthma Population (n): 40 Adults (age 18–75)	Educational program	Period: Screening at 0, 3, 6, and 12 months. Frequnecy: delivers quarterly and monthly telephone monitoring. Activity: five modules of educational contents (asthma pathophysiology, illness perceptions, medication skills, self-monitoring techniques, and environmental control and avoidance strategies), repeated at every visit.	Asthma exacerbation: significant reduction (from 2.80 to 0.58 episodes per patient per year) Medication adherence: improved over the time Pulmonary function: increased significantly (FEV1 from 59.2 to 66.6% of the predicted value) FeNO: decreased significantly Baseline: 36.0 ± 5.6 ppb; 6 months: 24.7 ± 3.2 ppb (*p* = 0.023), 12 months: 23.7 ± 3.6 ppb (*p* = 0.001) Quality of life: measured by ACQ-5, ASUI, AQLQ[S], after 6 months, revealed a significant improvement in perceived QoL Serum biomarkers: no significant change
Ricketts et al. (17, 18)	Randomized controlled trial+ follow-up of all patients	Uncontrolled asthma Adults (age 18–80), BMI ≥ 25Kg/m^2^ Population (n): 95 (48 Intervention Group[IG), 47 Usual care (UC)]	Pulmonary rehabilitation	Duration: 8 weeks Frequency: 1/week (1 h exercise, 1 h education) + 2 home based sessions Contents: Muscle resistance, aerobic training, educational program. V1:baseline, V2:after 8-weeks, V3:1-year follow-up	After 8 weeks program Quality of life: no significant between-group difference in change was observed, with only a non-significant trend toward improvement in the activity domain. Asthma control (ACQ6): significant improvement in the IG in both mean change and the proportion of patients achieving a clinically relevant improvement (mean change: −0.4 in IG vs. − 0.3 in UC) Dyspnoea (MRC): significant improvement in dyspnoea scores in the IG (Median change: 0 (− 1 to 0) in PR versus 0 (0–1) in UC, *p* = 0.022.) Exercise capacity (6MWD): increased walking distance (+20 m) in the IG, while the CG showed a reduction (−10 m). Breathlessness post-test (Borg scale) also improved in the IG. Other parameters: no relevant changes in anxiety/depression (HADS), body composition (BMI), inflammatory markers (eosinophils, FeNO) or lung function (spirometry). No measurable change in objectively assessed physical activity from accelerometry. After 1 year follow-up Asthma Control: significant improvements in ACQ-6 (mean (95% CI): V1:2.5 (2.1–2.9), V2:2.2 (1.8–2.5), V3: 2.3 (1.9–2.7); *p* = 0.003). Dyspnoea (MRC score): improvement in median score (median (IQR), V1: 3 (2–4); V2: 3 (2–3); V3: 3 (2–4); *p* = 0.010) Borg score after the longest 6MWT: improvement (median (IQR) V1:2 (0.5–3); V2:1 (0–2); V3:1 (0.5–2), *p* = 0.035). BMI: decreased slightly but significantly Maintenance OCS use: declined from 36 to 27% at one year. Asthma exacerbations requiring prednisolone: significantly reduced (median (IQR), V1:3 (2–5); V2:0 (0–4.7); V3 1.5 (0–4.2), p = 0.003) Urgent, unscheduled GP visits: significantly reduced (median (IQR), V1:2 (0–3.5); V2:0 (0–5.1); V3:0 (0–2.9), *p* = 0.025) No significant differences: AQLQ or its domains, Hospital Anxiety and Depression Scale (HADS), six-minute walk distance (6MWD), emergency department attendances or hospital admissions for asthma, or objectively measured physical activity.
Lanario et al. (35)	Feasibility study	Severe asthma Population (n): 28 (12 at the follow-up) Adults	Body reprogramming	Intervention: 4 sessions provided online, 30 min each Contents: introduction, stress and relaxation, movement and exercise, diet.	Quality of life (SAQ): consistent and potentially clinically meaningful improvement across participant. Asthma control (ACQ): overall improvement in asthma control but with greater variability in responses at follow-up. Extra-pulmonary symptom burden (GSQ-A): reduction in burden about extra-pulmonary symptoms. Emotional well-being (PANAS): overall, participants reported increased positive affect and reduced negative affect, suggesting improved mood and emotional wellbeing, albeit with more varied responses across individuals.

AQLQ, asthma quality of life questionnaire (AQLQ(S), standardized version); ACQ, asthma control questionnaire (ACQ-6, short-form; ACQ-5, symptoms only); FEV1, forced expiratory volume in 1 s; FVC, forced vital capacity; FRC, functional residual capacity; ERV, expiratory reserve volume; BMI, body mass index; HADS, hospital anxiety and depression scale; FEV1/FVC, modified Tiffeneau-Pinelli index (Forced Expiratory Volume in 1 s/ Forced Vital Capacity); IL-6, Interleukin 6; HIIT, high intensity interval training.

HR_max_, maximum heart rate max; HRQOL, health-related quality of life; SMD, standardized mean differences; MVV, maximum voluntary ventilation; 6MWT, six-minute walk test (6MWD, six-minute walk distance); QoL, quality of life; VO_2max_, maximum volume of oxygen; PI_max_, maximal inspiratory pressure; PE_max_, maximal expiratory pressure; IMT, inspiratory muscle training; PEF, peak expiratory flow; ISWT, Incremental shuttle walk test; SABA, short-acting beta agonists; PEFR, peak expiratory flow rate; GP, general practitioner; FeNO, fractional exhaled nitric oxide; ASUI, asthma symptom utility index; MRC, medical research council dyspnea scale; OCS, oral corticosteroids; SAQ, severe asthma questionnaire; GSQ-A, general symptom questionnaire-asthma; PANAS, positive and negative affect schedule.

### 4.1 Exercise training

Structured exercise training is the principal active component of PR. Aerobic training is safe in adults with asthma and is associated with improved symptom control, HRQoL (health related quality of life), maximal exercise capacity (VO₂max and HRmax) and functional capacity; benefits are more evident when exercise is combined with dietary, educational or behavioural interventions and performed at moderate intensity or above (10, 11, 20–22). Poor evidence is reported on a role played by exercise on inflammation in severe asthma: moderate-intense activity conducted for 12 weeks appears to reduce airway inflammation, but not at a systemic level (22). However, in patients with early-stage asthma, including mild intermittent asthma, there is promising evidence that moderate-intensity supervised aerobic training (SAT), performed three times per week, decreases pulmonary and systemic profibrotic biomarkers while increasing antifibrotic markers, and its anti-inflammatory effect is sensitively detected by FeNO. It also reduces airway eosinophil and macrophage infiltration, as well as peripheral blood eosinophil and lymphocyte counts (23). Quality of life (AQLQ) and asthma control questionnaire (ACQ) showed improvement as a result of moderate- and vigorous-intensity training (24). Resistance training mitigates muscle mass and strength loss, reduces fall risk and exertional dyspnoea compared to sustained high-intensity continuous exercise, improving tolerance and enabling progressive load increases (8). Interval training alternating high-intensity bouts with recovery periods is suitable where dyspnoea or fatigue limit longer continuous efforts, supporting higher training stimulus with lower perceived symptoms (8). Walking aids including adjunctive oxygen support when necessary may assist severely impaired patients by improving respiratory mechanics (25). Use of fitness trackers is recommended for objective monitoring and behaviour support (26), while family involvement, and patient network engagement enhance adherence (27, 28).

### 4.2 Neuromuscular electrical stimulation (NMES)

NMES is an adjunctive option to strengthen peripheral muscle without active exertion and is particularly useful in highly debilitated patient (4). Recent trials in older adults with mild persistent asthma reported that adding NMES to conventional rehabilitation improved quadriceps strength, endurance, functional capacity, dyspnoea and HRQoL within four weeks, with a favourable safety profile (29).

### 4.3 Inspiratory muscle training (IMT)

Airflow obstruction and dynamic hyperinflation increase inspiratory workload and promote diaphragm flattening, reducing mechanical efficiency. IMT, applied using threshold or tapered resistive devices, increases maximal inspiratory pressures (PImax), endurance and reduces dyspnoea when training intensity reaches or exceeds approximately 30% of PImax. IMT was found to be most beneficial in patients with documented inspiratory muscle weakness (PImax or PEmax below ~65–80% predicted); in these cases, incremental benefits can be gained by whole-body training are most evident in these cases (29–31).

### 4.4 Breathing retraining and adjunctive interventions

Breathing retraining methods (Buteyko, Papworth, yoga-based techniques) target dysfunctional breathing patterns, improving symptom perception, HRQoL and psychological outcomes although effects on lung function and exacerbation frequency are limited (32–34). Adjunctive supports such as bronchodilator therapy optimisation, oxygen supplementation, anabolic support, heliox or non-invasive ventilation may enhance exercise tolerance for selected patients but require individualised risk–benefit assessment (8).

### 4.5 Education and self-management

Education is a core component of PR and is recommended within GINA algorithms for difficult-to-treat asthma (1). Contemporary educational strategies emphasize shared decision-making, problem-solving and self-management skills, addressing medication safety (including biologic therapy and corticosteroid use), exacerbation action plans, inhaler technique and lifestyle counselling (8, 12, 28). Integrated educational modules within PR improve self-efficacy and may enhance adherence, though evaluating the independent effect of education is methodologically challenging (17, 35–37).

### 4.6 Telerehabilitation

Telerehabilitation offers flexible, incremental delivery—ranging from synchronous supervised sessions to asynchronous educational modules, exercise videos and symptom tracking—and can broaden access for patients with mobility constraints or geographic barriers (38). Hybrid models including initial in-person assessment followed by remote supervision may optimise safety, engagement and cost-efficiency. Challenges include variable digital literacy, technology access and the need to ensure intervention fidelity.

### 4.7 Maintenance strategies and outcomes

Effective maintenance strategies have demonstrated benefit include periodic supervised booster sessions, telephone or text-message support combined with structured home exercise, diaries and activity monitors; these approaches can sustain walking distance and activity, though evidence for preserved HRQoL is not consistent (39). Optimal contact frequency is uncertain; follow-up intervals vary among programmes, with some adopting quarterly reviews and others semi-annual monitoring after the first year (18, 40). Economic analyses and cost-effectiveness data remain scant, with variability across health systems. Core outcome domains include HRQoL, asthma control (ACQ/ACT), exercise capacity (6MWT: Six-minute Walk test, 6MST), objectively measured physical activity, anxiety and depression, respiratory function, inflammatory biomarkers and medication adherence. Long-term follow-up is desirable because PR benefits may reduce within 6–12 months without maintenance (8, 9); however standardized timing criteria remain undefined.

### 4.8 Key performance indicators

Standardized process and performance indicators facilitate benchmarking and quality assurance. Suggested metrics include clinical outcomes (control scores, exacerbation and hospitalization rates), functional measures (6MWT, exercise tolerance), psychological outcomes, healthcare utilization and cost-effectiveness indicators to support service evaluation and commissioning (9, 13, 41, 42) (Table 2).

**Table 2:** Performance indicators base on clinical, functional, economic and organizational scope and timing to measure them (9, 10, 39, 40).

Scope	Performance indicators	How to measure	When to measure
Clinical	Improvement in: ACT AQLQ Spirometry measures	Validated test	Baseline, after 8-weeks program, follow-up at 3–6-12 months
Functional	6MWT Daily physical activity level Program Adherence Behavioral changes	Functional test Digital instruments Reported measures/anamnestic assesment	Baseline, after 8-weeks program, follow-up at 3–6-12 months
Economic	Number of exacerbations/year Number of hospitalization/year Change in work productivity (days lost per year) Funding stability QALY	Medical chart+anamnestic assessment Management audit	Annual
Organizational	Number of patients enrolled in the program % of follow-up completed	Patient’s register	Continuos

ACT, asthma control test; AQLQ, asthma quality of life questionnaire; 6MWT, six-minute walk test; QALY, cost per quality-adjusted life year.

## 5 Discussion

Severe and uncontrolled asthma presents ongoing challenges despite pharmacological advances. PR offers a multidimensional strategy addressing deconditioning, dysfunctional breathing, multimorbidity and self-management deficits. Evidence indicates that structured exercise, particularly when combined with education and behavioural support, improves exercise capacity, dyspnoea and HRQoL and may reduce exacerbation burden in selected cohorts (18–22). IMT delivers important gains in inspiratory strength and symptom relief when respiratory muscle weakness is present (30, 31). Breathing retraining is effective on symptom perception and psychological measures but does not improve pulmonary function test spirometry nor reduce exacerbation risk substantially (32–34, 43, 44). Biking is safe, adaptable and appropriate for patients with osteopenia or osteoporosis, which are common comorbidities in severe asthma (45).

Referral criteria should consider persistent symptoms, functional limitation, frequent exacerbations and healthcare utilization as well as abnormal pulmonary function test; contraindications are uncommon and most barriers can be addressed by program adaptation (8).

Program heterogeneity and small study sample sizes limited the strength of recommendations and generalisability. High dropout indicates to the need for flexible, patient-centred delivery models that may combine employment commitments and individual preferences; hybrid programmes, evening or weekend sessions and telerehabilitation options may improve access and retention (13, 17, 22). Adherence is supported by engaging, varied exercise prescriptions, fitness trackers, family involvement and educational initiatives that increase perceived benefits and self-efficacy (26–28). Safety considerations include cardiovascular and musculoskeletal screening, attention to corticosteroid-related risks and clear procedures for managing exacerbations during rehabilitation programmes (2, 8). In particular it is known that Exercise-induced bronchoconstriction (EIB) may occur in otherwise well-controlled asthma, typically 5–10 min post-exercise, with symptoms such as dyspnea, wheeze, chest tightness, or cough. GINA guidelines recommend pre-medicate with a rapid-acting inhaled b2-agonist prior to exercise, with leukotriene receptor antagonists or cromones as alternatives, and include a gradual warm-up (1, 46).

From an economic standpoint, PR may be cost-effective by preventing escalation of pharmacotherapy, reducing unplanned visits and hospital admissions and improving productivity through reduced absenteeism and presenteeism. PR shows a higher QALY (Cost per quality-adjusted life year) gain compared to other treatments (9, 47). Rigorous health-economic analyses are needed to guide policymakers.

Additional considerations include the high prevalence of comorbid conditions—chronic rhinosinusitis, obesity, gastroesophageal reflux and psychiatric disorders—which may influence rehabilitation needs and outcomes and should be proactively managed within multidisciplinary programmes (5). Multidisciplinary team commonly includes respiratory physicians, physiotherapists/exercise physiologists, specialist nurses, psychologists and dietitians; competency frameworks and training are required to ensure safe, standardised delivery (9).

Scientific evidence highlights the importance of fidelity and adherence metrics; trials should prespecify adherence thresholds and report intention-to-treat effects. Standardised reporting of intervention dose, intensity and adherence will enable meta-analysis and possibility to identify active strategies.

Technological supports including wearables, smartphone apps and secure telehealth platforms may enable objective monitoring, remote supervision and patients’education, yet digital inequities and data governance require attention (22, 38, 39). In order to disseminate rehabilitation in severe asthma, efforts should be done to address equity, ensuring educational materials and community delivery options to underserved populations who bear disproportionate asthma morbidity.

## 6 Conclusion

Pulmonary rehabilitation represents a promising adjunctive intervention for patients with severe or uncontrolled asthma. Multidimensional programmes that combine exercise training, education and behavioural support produce clinically relevant improvements in asthma control, dyspnoea, exercise capacity and quality of life in selected populations. However, the evidence remains heterogeneous and limited in severe asthma patients. Future priorities include large, phenotype-stratified randomized trials, harmonised core outcome sets including patient-reported and economic endpoints, standardisation of programme and implementation of research to determine optimal delivery models and cost-effectiveness. Recommendations and future directions include adopting a personalised, phenotype-informed approach to rehabilitation referral and design, with early identification of patients who are deconditioned, physically inactive, or experiencing frequent exacerbations despite optimal pharmacotherapy. Multidisciplinary teams should tailor interventions to address comorbid contributors such as obesity, psychological distress and sinonasal disease, and should incorporate objective activity monitoring, stratified goal setting, and family or community support to promote long-term behaviour change. Programmes should also consider occupational and social determinants of health and offer flexible scheduling and hybrid delivery models. Safety screening, inhaler technique optimisation, action plans and integration with severe asthma Clinics are essential components. Shared core outcome sets and registries will facilitate pooled analyses and benchmarking. Education plans should be standardised across programmes while allowing local adaptation. Funding should prioritise pragmatic trials and implementation research, and healthcare systems should consider reimbursement mechanisms to support multidisciplinary rehabilitation teams. Ultimately, embedding PR within strategic approach of severe asthma Centers may optimise patient-centred outcomes and reduce healthcare utilisation.
